# Mid-term clinical outcomes of left bundle branch area pacing compared to accurate right ventricular septal pacing

**DOI:** 10.1007/s10840-024-01890-z

**Published:** 2024-07-29

**Authors:** Yousaku Okubo, Takumi Sakai, Shogo Miyamoto, Yukimi Uotani, Naoto Oguri, Motoki Furutani, Shunsuke Miyauchi, Sho Okamura, Takehito Tokuyama, Yukiko Nakano

**Affiliations:** Department of Cardiovascular Medicine, Hiroshima University Graduate School of Biomedical and Health Sciences, Hiroshima, Japan

**Keywords:** Conduction system pacing, Left bundle branch area pacing, Atrioventricular block, Right ventricular septal pacing, Delivery catheter

## Abstract

**Background:**

Although left bundle branch area pacing (LBBAP) reportedly results in fewer adverse outcomes after implantation than conventional stylet-guided right ventricular septal pacing (RVSP), previous studies have not compared LBBAP with accurate RVSP using a delivery catheter. The aim of this study was to compare clinical outcomes between LBBAP and accurate RVSP among patients with atrioventricular block (AVB).

**Methods:**

This single-center observational study enrolled 160 patients requiring RV pacing due to symptomatic AVB between September 2018 and December 2021. Primary composite outcomes included all-cause death, hospitalization due to heart failure (HF), and upgrading to biventricular pacing. Secondary composite outcomes included any procedural and postprocedural complications.

**Results:**

Overall, 160 patients were analyzed (LBBAP, *n* = 81; RVSP, *n* = 79). No significant differences in baseline characteristics were observed between the two groups. The RV pacing burden at 1 year after implantation was 90.8% ± 20.4% and 86.2% ± 22.6%, respectively (*p* = 0.21). During a mean follow-up of 840 ± 369 days, the incidence of the primary outcome was significantly lower with LBBAP (4.9%) compared to RVSP (22.8%) (Log-rank *p* = 0.02). There was no significant difference in the incidence of the secondary outcome between the two groups (3.7% vs. 5.1%, *p* = 0.65). In the multivariate analysis, baseline QRS duration, RV pacing burden, and LBBAP were independently associated with the primary outcome (baseline QRS duration: hazard ratio [HR], 1.01; 95% confidence interval [CI], 1.00–1.02; *p* < 0.001; RV pacing burden: HR, 1.01; 95% CI, 1.00–1.02; *p* < 0.001; LBBAP: HR, 0.45; 95% CI, 0.31–0.64; *p* < 0.001).

**Conclusion:**

In patients requiring frequent RV pacing, LBBAP was associated with reduced adverse clinical outcome compared to accurate RVSP using a delivery catheter.

**Graphical Abstract:**

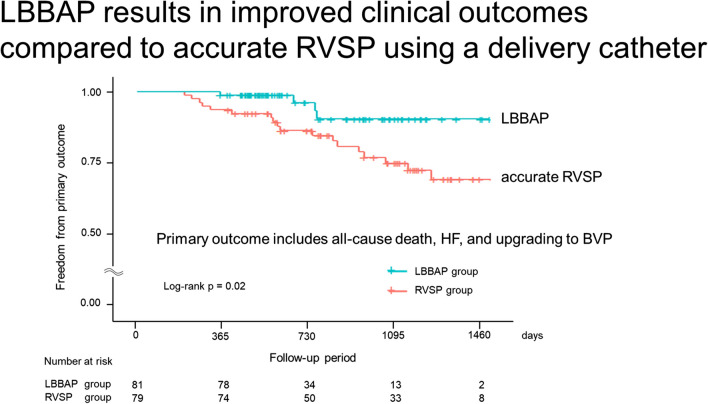

**Supplementary Information:**

The online version contains supplementary material available at 10.1007/s10840-024-01890-z.

## Introduction

Left bundle branch area pacing (LBBAP) is a pacing method that can maintain physiological ventricular activation by capturing the conduction system around the area of the LBB [1–3]. Reports have exhibited a significantly lower incidence of composite adverse outcomes, including death, hospitalization due to heart failure (HF), and upgrading to biventricular pacing (BVP), in the LBBAP group compared to the conventional right ventricular (RV) pacing group [4, 5]. However, a conventional stylet-guided lead was used in these studies, and it remains uncertain whether conventional RV septal pacing (RVSP) was reliably implanted in the RV septum. Sometimes, conventional stylet-guided RV lead implantation may not achieve accurate RVSP. Hattori et al. reported that, in some cases, the RV lead is implanted in the free wall instead of the septum when using a conventional stylet-guide technique, resulting in a worse prognosis compared with accurate RVSP [6]. Recently, delivery catheters have become available for pacemaker lead implantation, making it easier to accurately place the leads in the RV septum. A randomized control study reported that catheter-guided RV lead implantation could achieve more accurate RV lead placement in the RV septum, as assessed by computed tomography, and a narrower paced-QRS duration than the traditional stylet-based technique [7]. However, to the best of our knowledge, there are no studies comparing the clinical outcomes of accurate RVSP performed with delivery catheter guide alone, not stylet guide, with those of LBBAP. Therefore, this study aimed to compare the clinical outcomes between LBBAP and accurate RVSP through a delivery catheter in patients requiring frequent RV pacing due to atrioventricular block (AVB).

## Methods

### Study design and patient population

This single-center, prospective, nonrandomized, controlled clinical study included 196 consecutive patients who required frequent RV pacing due to symptomatic complete or high-degree AVB at Hiroshima University Hospital between September 2018 and December 2021. Patients who required an implantable cardioverter defibrillator (ICD) or cardiac resynchronized therapy (CRT) were excluded. All patients in this study underwent either LBBAP or accurate RVSP using a delivery catheter according to operator preference. This study was approved by the Ethics Committee of the Hiroshima University Hospital, and all participants provided written informed consent.

### Procedure

All procedures were performed using the Select Secure 3830 pacing lead (Medtronic Inc., Minneapolis, MN, USA) and fixed-curve C315 His sheath (Medtronic Inc., Minneapolis, MN, USA). The delivery sheath was advanced into the RV over a guidewire though the axillary vein. In the LBBAP group, we first mapped the region where the His bundle electrogram could be recorded. Thereafter, the tip of the sheath was moved 1.5–2 cm toward the RV apex from the His bundle location and rotated counterclockwise to perpendicularly contact the long axis of the interventricular septum. Subsequently, paced-QRS morphology was evaluated by unipolar pacing. The lead was carefully screwed at the site where the paced-QRS morphology showed a “W” pattern with a notch in lead V1. During the procedure, the lead depth inside the RV septum was measured via angiography with contrast medium injection from the sheath under the left anterior oblique (LAO) view at 30°. While the lead was advanced, paced-QRS morphology and lead impedance were measured intermittently. Unipolar impedance with a sudden drop (> 200 ohms) or < 500 ohms may indicate that the tip of the lead was exposed to the LV cavity. In such cases, the pacing lead was repositioned to another site. LBBAP was considered successful if the pacing lead was located deep in the RV septum based on the following criteria: (1) paced-QRS morphology of right bundle branch conduction delay (qR, QR, Qr, or rSR) pattern during unipolar pacing; (2) short (< 80 ms) and stable paced-LV activation time (LVAT) in leads V5–V6 during threshold testing; and (3) demonstration of transition from nonselective to selective LBBP/left ventricular septal pacing (LVSP) during threshold testing. In the RVSP group, all leads were confirmed to be screwed in the RV septum by angiography with contrast medium injection from the sheath. Lead parameters, including the pacing capture threshold, R-wave amplitude, and lead impedance, were measured when the bottom of the lead helix reached the RV septum. If the lead parameters were acceptable, the lead was not advanced any further and was fixed. In the RVSP group, the region of the implanted lead was assessed. An apical septal region was defined as the bottom of the right ventricle close to the apex, or at the inferior third of the interventricular septum. A high septal region was defined as the upper third of the interventricular septum (outflow tract region). A mid septal region was defined as the area between the upper third of the interventricular septum (high septum) and the inferior third of the same (apical septum). In both groups, the atrial lead was screwed at the right atrial septal wall or placed within the right atrial appendage.

### Device programming

All patients received a dual-chamber pacemaker. After implantation, the pacemaker was programmed to the DDD(R) mode with an initial basic pacing rate of 60–70 bpm. Atrioventricular delay was programmed at 120 ms for sensed P waves and 150 ms for paced atrial activation.

### Data collection and follow-up

Baseline clinical characteristics and medical history were obtained from medical records on hospital admission. Transthoracic echocardiography was performed at hospital admission and after 1 year of follow-up. LV ejection fraction (LVEF), LV end-diastolic dimension, LV systolic dimension, interventricular septum thickness, and tricuspid regurgitation (TR) grade were measured by experienced echocardiographers who were blinded to the clinical data. According to the American Society of Echocardiography, TR was categorized into none or trace, mild, moderate, and severe. The severity of TR was classified as grade 1 (none or trace), grade 2 (mild), grade 3 (moderate), and grade 4 (severe). The change in TR of one grade was defined as worsening or improving TR grade by ≥ one grade at 1 year after pacemaker implantation compared with baseline. Blood samples for laboratory testing were collected from a peripheral vein at baseline and at follow-up 1 year after pacemaker implantation. Electrocardiographic parameters were measured on hospital admission and at the 1-year follow-up. Paced LVAT was defined as the interval from the pacing stimulus to the R-wave peak of the QRS complex in leads V5–V6. QRS duration and paced-QRS duration were measured in all leads and were defined as the interval from the end of the PR interval and the pacing stimulus, respectively, to the end of the S-wave. Pacing lead parameters, including amplitude, threshold, and impedance, were measured in VVI mode and were assessed during the procedure and at the 1-year follow-up.

### Primary and secondary composite outcomes

The primary composite outcome combined all-cause death, hospitalization due to HF, and upgrading to BVP. The secondary composite outcome combined procedural and postprocedural complications such as pneumothorax, device infection, lead perforation, and lead revision during follow-up.

### Statistical analysis

Continuous variables are presented as mean ± standard deviation or median with interquartile range (25th–75th percentiles), and categorical variables are presented as proportions. Descriptive statistics were used to describe the baseline characteristics in each group with *χ*^2^ tests for binomial and categorical data, unpaired 2-tailed *t* tests for normally distributed continuous variables, and the Mann–Whitney test for skewed continuous variables. The log-rank test was used to compare event-free survivals. Cox proportional hazard models were used to estimate survival probability for the composite primary outcomes between the LBBAP and accurate RVSP group, which were presented as hazard ratios (HR) and 95% confidence intervals (CI). Statistically significant variables in the univariate analysis were included in multivariate models. Due to the relatively low number of primary outcome events, we employed covariate adjustment using the propensity score for multivariate modeling. For instance, in assessing the adjusted effect of LBBAP, we created a propensity score that included other confounding factors such as baseline LVEF, baseline QRS duration, and RV pacing burden. This propensity score was then utilized as a covariate in the model to evaluate the adjusted effect of each factor. All analyses were performed using R4.3.1, with *p* < 0.05 indicating statistical significance.

## Results

### Baseline clinical characteristics

**Fig. 1:**
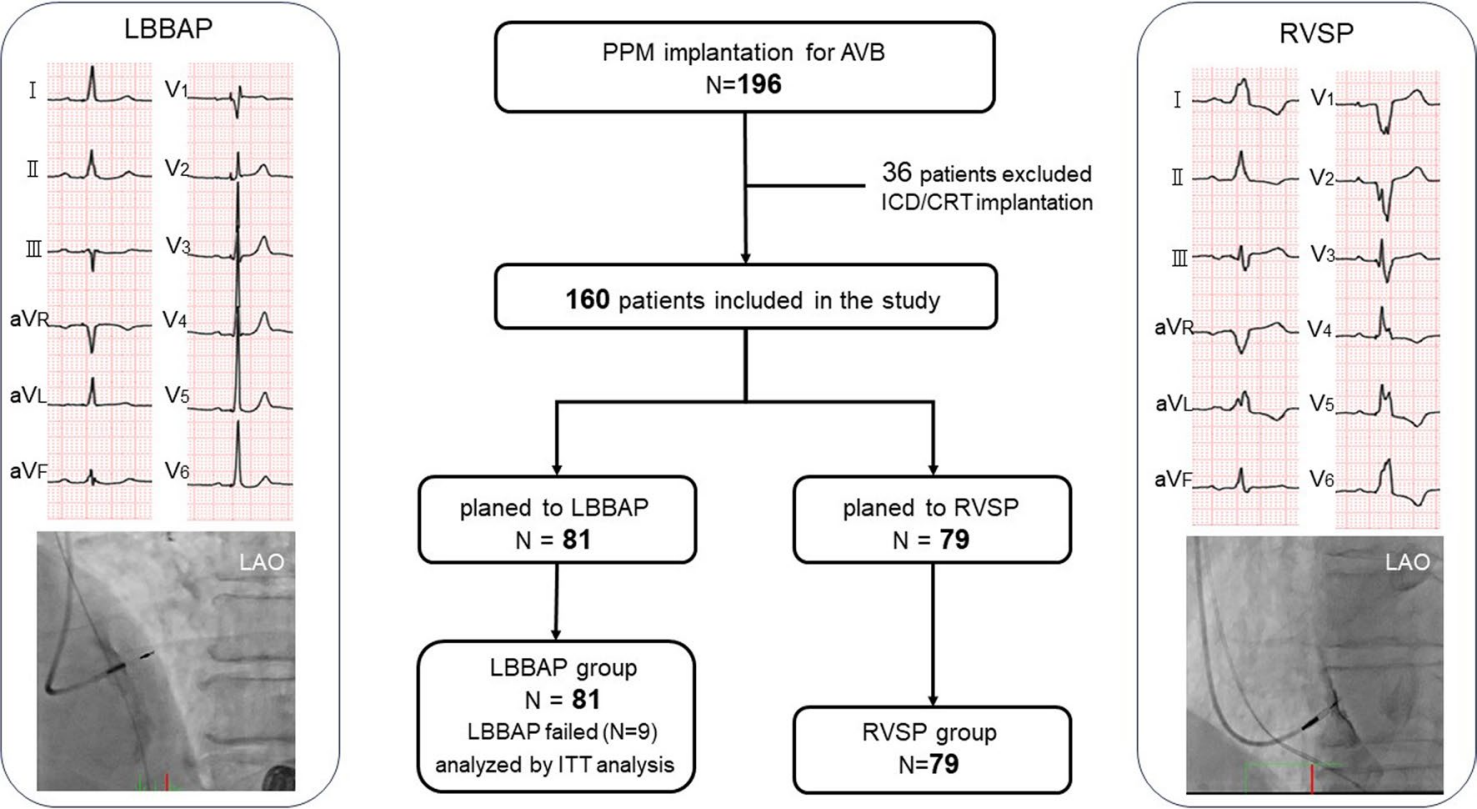
Study design and patient population. PPM, permanent pacemaker; AVB, atrioventricular block; RVSP, right ventricular septal pacing; LBBAP, left bundle branch area pacing; ICD, implantable cardioverter defibrillator; CRT, cardiac resynchronization therapy; ITT, intention to treat

Overall, 196 consecutive patients underwent permanent pacemaker implantation due to symptomatic complete or high-degree AVB during the study period. Patients who required ICD or CRT were excluded (*N* = 36); 160 patients met the final inclusion criteria. LBBAP was successful in 72 (88.8%) patients. Nine patients in whom LBBAP could not be achieved had a deep septal pacing in which the lead was placed deep into the RV septum. These 9 patients remain in the LBBAP group for intention-to-treat analysis. Thus, 160 patients were enrolled and prospectively followed for at least 1 year after pacemaker implantation. Among these patients, 81 underwent LBBAP, while 79 underwent accurate RVSP (Fig. 1).

Baseline clinical characteristics of all patients are presented in Table 1. The mean age of the entire cohort was 77.2 ± 11.0 years, 87 (54.3%) patients were men, 70.0% had complete AVB, 51.9% had a history of HF, and 36.3% had a history of paroxysmal AF. The mean baseline LVEF and QRS duration were 58.9 ± 9.1% and 110.8 ± 26.3 ms, respectively. Overall, 94 (58.8%) patients had organic heart disease; a third of whom had valvular heart disease and nonischemic cardiomyopathy. No significant differences in baseline characteristics were observed between the two groups.

**Table 1:** Baseline clinical characteristics of participants

Variables	Overall	LBBAP	RVSP	*p* value
	*n* = 160	*n* = 81	*n* = 79	
Age, years	77.2 ± 11.0	76.9 ± 11.7	77.4 ± 10.3	0.78
Male, *n* (%)	87 (54.3)	40 (49.4)	47 (59.5)	0.19
Advanced AVB, *n* (%)	48 (30.0)	21 (25.9)	27 (34.2)	0.12
Complete AVB, *n* (%)	112 (70.0)	60 (74.0)	52 (65.8)	0.12
Hypertension, *n* (%)	121 (75.6)	63 (77.8)	58 (73.4)	0.52
Dyslipidemia, *n* (%)	63 (39.4)	28 (34.6)	35 (44.3)	0.21
Diabetes, *n* (%)	39 (24.4)	20 (24.7)	19 (24.1)	0.92
Prior stroke, *n* (%)	17 (10.6)	8 (9.9)	9 (11.4)	0.75
Previous MI, *n* (%)	15 (9.4)	7 (8.6)	8 (10.1)	0.56
Prior heart failure, *n* (%)	83 (51.9)	38 (46.9)	45 (56.9)	0.20
Paroxysmal atrial fibrillation, *n* (%)	58 (36.3)	29 (35.8)	29 (36.7)	0.90
Ischemic cardiomyopathy, *n* (%)	20 (12.5)	7 (8.6)	13 (16.5)	0.13
Non-ischemic cardiomyopathy, *n* (%)	40 (25.0)	19 (23.5)	21 (26.6)	0.64
Valvular heart disease, *n* (%)	34 (21.2)	16 (19.8)	18 (22.8)	0.64
QRS duration, ms	110.8 ± 26.3	113.7 ± 26.3	107.8 ± 26.1	0.16
LBBB morphology, *n* (%)	32 (20.0)	16 (19.8)	16 (21.1)	0.94
RBBB morphology, *n* (%)	35 (21.9)	21 (25.9)	14 (17.8)	0.21
LVEF, %	58.9 ± 9.1	58.8 ± 9.5	59.1 ± 8.7	0.84
LVDd, mm	48.2 ± 5.5	48.4 ± 5.8	47.9 ± 5.3	0.64
LVDs, mm	33.0 ± 6.6	33.1 ± 6.9	32.9 ± 6.4	0.93
IVS, mm	9.2 ± 1.7	9.0 ± 1.4	9.4 ± 2.0	0.18
TR grade 0	44 (27.5)	18 (22.2)	26 (32.9)	0.38
TR grade 1	97 (60.6)	52 (64.2)	45 (56.9)	
TR grade 2	15 (9.4)	8 (9.9)	7 (8.0)	
TR grade 3	4 (2.5)	3 (3.7)	1 (1.3)	
TR grade 4	0 (0.0)	0 (0.0)	0 (0.0)	
Hemoglobin, g/dl	12.8 ± 1.8	12.8 ± 1.9	12.8 ± 1.7	0.92
Blood urea nitrogen, mg/dl	20.9 ± 8.3	21.3 ± 8.6	20.5 ± 8.0	0.55
Serum creatinine, mg/dl	1.1 ± 0.7	1.1 ± 0.8	1.0 ± 0.6	0.69
NT-proBNP, pg/ml	1097.4 (245.0–1377.0)	1310.8 (270.7–1565.0)	878.9 (240.0–1139.0)	0.12
ACE-I or ARB, *n* (%)	114 (71.3)	57 (70.4)	57 (74.0)	0.60
β-Blocker, *n* (%)	66 (41.3)	29 (35.8)	37 (48.1)	0.12
Diuretic, *n* (%)	66 (41.3)	33 (40.7)	33 (41.8)	0.89

*LBBAP*, left bundle branch area pacing; *RVSP*, right ventricular septal pacing; *AVB*, atrioventricular block; *MI*, myocardial infarction; *LBBB*, left bundle branch block; *RBBB*, right bundle branch block; *LVEF*, left ventricular ejection fraction; *LVDd*, left ventricular end-diastolic diameter; *LVDs*, left ventricular systolic diameter; *IVS*, interventricular septum thickness; *TR*, tricuspid regurgitation; *NT-proBNP*, N-terminal pro-B type natriuretic peptide; *ACE-I*, angiotensin converting enzyme inhibitor; *ARB*, angiotensin II receptor blocker

### Procedural and clinical outcomes during follow-up

Procedural and clinical outcomes during follow-up are presented in Tables 2 and 3. The mean procedural and fluoroscopic times were significantly longer in the LBBAP group than in the RVSP group (128.7 ± 20.7 min vs. 113.7 ± 15.6 min, *p* < 0.001; 15.9 ± 4.8 min vs. 12.1 ± 3.4 min, *p* < 0.001, respectively). In the RVSP group, the proportions of each lead positions were as follows; high septum = 8 (10.1%), mid septum = 64 (81.0%), and low septum = 7 (8.9%).

**Table 2:** Clinical and lead parameters at baseline and follow-up and procedural characteristics

Variables	LBBAP	RVSP	*p* value
	*n* = 81	*n* = 79	
Procedure time, min	128.7 ± 20.7	113.7 ± 15.6	< 0.001
Fluoroscopic time, min	15.9 ± 4.8	12.1 ± 3.4	< 0.001
Paced QRS duration, ms	123.8 ± 12.8	149.5 ± 12.8	< 0.001
Paced LVAT, ms	68.4 ± 13.8	93.2 ± 14.7	< 0.001
Lead parameter at implantation			
Pacing capture threshold, V/0.4 ms	0.7 ± 0.2	0.8 ± 0.3	0.01
R wave amplitude, mV	17.4 ± 4.7	12.4 ± 4.8	< 0.001
Lead impedance, Ω	589.3 ± 98.2	587.3 ± 111.6	0.87
Lead parameter at 1-year follow-up			
Pacing capture threshold, V/0.4 ms	0.9 ± 0.3	1.1 ± 0.3	< 0.001
R wave amplitude, mV	16.4 ± 4.5	12.3 ± 5.0	< 0.001
Lead impedance, Ω	589.5 ± 86.3	598.5 ± 101.9	0.82
RV pacing burden, %	90.8 ± 20.4	86.2 ± 22.6	0.21
LVEF, %	59.1 ± 8.7	55.4 ± 10.8	0.03
LVDd, mm	47.6 ± 4.8	49.2 ± 6.4	0.09
LVDs, mm	32.3 ± 6.7	34.8 ± 7.1	0.04
TR grade 0	20 (24.7)	23 (29.1)	0.37
TR grade 1	50 (61.7)	39 (49.3)	
TR grade 2	7 (8.6)	14 (17.7)	
TR grade 3	2 (2.5)	1 (1.3)	
TR grade 4	2 (2.5)	2 (2.5)	
NT-proBNP, pg/ml	564.6 (107.6–633.5)	1007.5 (259.0–1501.3)	0.01

*LBBAP*, left bundle branch area pacing; *RVSP*, right ventricular septal pacing; *LVAT*, left ventricular activation time; *LVEF*, left ventricular ejection fraction; *LVDd*, left ventricular end-diastolic diameter; *LVDs*, left ventricular systolic diameter; *TR*, tricuspid regurgitation; *NT-proBNP*, N-terminal pro-B type natriuretic peptide

**Table 3:** Clinical outcomes

Variables	LBBAP	RVSP	*p* value
	*n* = 81	*n* = 79	
Primary composite outcome, *n* (%)	4 (4.9)	18 (22.8)	0.001
All cause death, *n* (%)	3 (3.7)	9 (11.4)	0.06
Hospitalization due to HF, *n* (%)	3 (3.7)	12 (15.2)	0.01
Upgrading to BVP, *n* (%)	0 (0.0)	2 (2.5)	0.10
Secondary composite outcome, *n* (%)	3 (3.7)	4 (5.1)	0.65
Lead perforation, *n* (%)	1 (1.2)	0 (0.0)	0.24
Lead revision, *n* (%)	1 (1.2)	2 (2.5)	0.38
Pneumothorax, *n* (%)	1 (1.2)	1 (1.3)	0.88
Device infection, *n* (%)	0 (0.0)	1 (1.3)	0.11

*LBBAP*, left bundle branch area pacing; *RVSP*, right ventricular septal pacing; *HF*, heart failure; *BVP*, biventricular pacing

The paced-QRS duration and paced LVAT were significantly shorter in the LBBAP group than in the RVSP group (123.8 ± 12.8 ms vs. 149.5 ± 12.8 ms, *p* < 0.001; 68.4 ± 13.8 ms vs. 93.2 ± 14.7 ms, *p* < 0.001, respectively). R-wave amplitude in the LBBAP group was significantly higher than that in the RVP group (17.4 ± 4.7 mV vs. 12.4 ± 4.8 mV, *p* < 0.001). The LBBAP group had a significantly lower pacing capture threshold compared to the RVSP group at implantation (0.7 ± 0.2 V/0.4 ms vs. 0.8 ± 0.3 V/0.4 ms, *p* < 0.001). The difference in R-wave amplitude and pacing capture threshold between the two groups after the procedure did not change at the 1-year follow-up (16.4 ± 4.5 mV vs. 12.3 ± 5.0 mV, *p* < 0.001; 0.9 ± 0.3 V/0.4 ms vs. 1.1 ± 0.3 V/0.4 ms, *p* < 0.001, respectively).

**Fig. 2:**
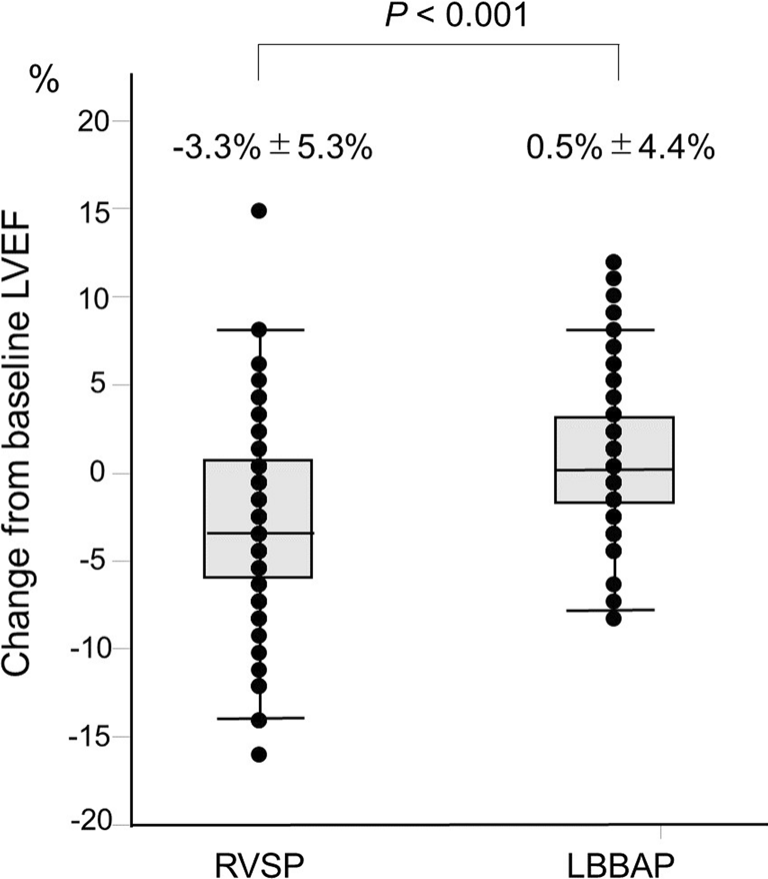
Changes from baseline LVEF between the two groups. There was a statistically significant difference in the mean percentage change from baseline LVEF between the RVSP group and the LBBAP group (− 3.3 ± 5.3% vs. 0.5 ± 4.4%, *p* < 0.001). LVEF, left ventricular ejection fraction; RVSP, right ventricular septal pacing; LBBAP, left bundle branch area pacing

During the 1-year follow-up, no significant difference in the RV pacing burden was observed between the groups (90.8 ± 20.4% vs. 86.2 ± 22.6%, *p* = 0.21). LVEF in the LBBAP group was significantly higher than that in the RVSP group at the 1-year follow-up (59.1 ± 8.7% vs. 55.4 ± 10.8%, *p* = 0.03). There was a significant difference in the mean percentage change from baseline LVEF between the RVSP group and LBBAP group (− 3.3% ± 5.3% vs. 0.5 ± 4.4%, *p* < 0.001; Fig. 2). The proportions of the TR grade at baseline and 1-year follow-up are presented in Table 1 and Table 2, respectively. The percentage of patients with worsening TR in the LBBAP group was significantly lower than that in the RVSP group (7.4% vs. 17.7%, *p* = 0.04; Supplementary Fig. 1). The N-terminal pro-B type natriuretic peptide level in the LBBAP group was significantly lower than that in the RVSP group at the 1-year follow-up (564.6 pg/mL [107.6–633.5] vs. 1007.5 pg/mL [259.0–1501.3], *p* = 0.01).

**Fig. 3:**
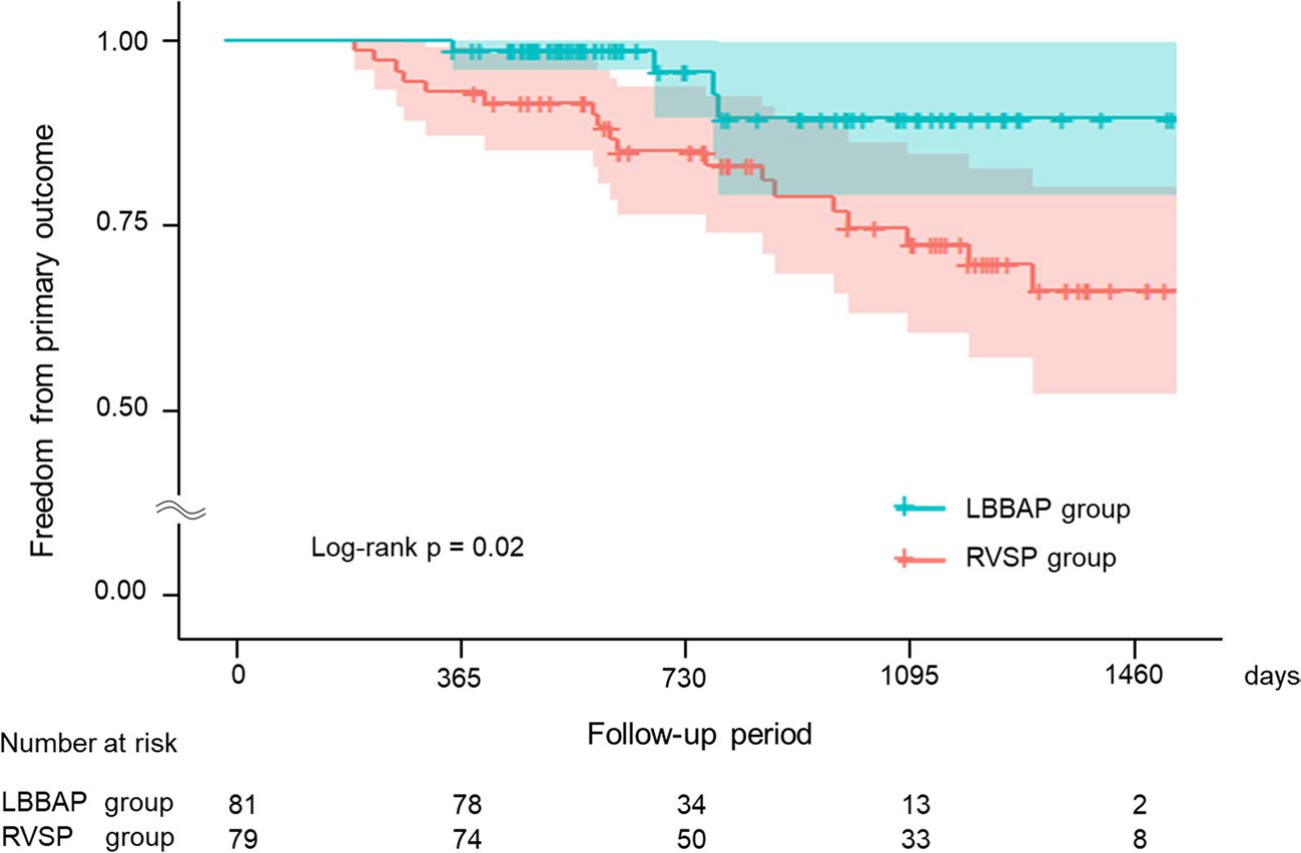
Kaplan–Meier survival curves and analysis for the primary outcome. Patients in the RVSP group were more likely than patients in the LBBAP group to develop the primary composite outcomes (all-cause death, hospitalization due to heart failure, and upgrading to biventricular pacing) (22.8% vs. 4.9%; Log-rank *p* = 0.02.). LBBAP, left bundle area pacing; RVSP, right ventricular septal pacing

During a mean follow-up period of 840 ± 369 days, the primary composite outcome occurred in 22 (13.8%) patients. Patients in the RVSP group were more likely than those in the LBBAP group to develop the primary outcome (22.8% vs. 4.9%; Log-rank *p* = 0.02) (Fig. 3). In the RVSP group, two patients upgraded to biventricular pacing (BVP). Both of them had no known cardiomyopathy before pacemaker implantation and had preserved LVEF. However, during follow-up, their LVEF decreased by more than 10% from baseline, and they experienced hospitalization due to heart failure. After alternative causes of cardiomyopathy were excluded, an additional LV lead was implanted.

In the multivariate analysis, the baseline QRS duration, RV pacing burden, and LBBAP were independently associated with the primary outcome (Table 4; baseline QRS duration: HR, 1.02; 95% CI, 1.00–1.02; *p* < 0.001; RV pacing burden: HR, 1.02; 95% CI, 1.00–1.03; *p* < 0.001; LBBAP: HR, 0.41; 95% CI, 0.29–0.58; *p* < 0.001).

**Table 4:** Cox proportional hazards for composite primary outcome

Variables	Unadjusted HR (95% CI)	*p* value	Adjusted HR (95% CI)	*p* value
Age, 1 years	1.00 (0.99–1.02)	0.39		
Sex, male	1.02 (0.73–1.44)	0.89		
Atrial fibrillation	1.13 (0.79–1.61)	0.50		
Prior HF	1.32 (0.94–1.85)	0.11		
Baseline LVEF	1.02 (1.00–1.04)	0.04	1.42 (0.55 –3.81)	0.47
Baseline QRS duration	1.02 (1.01–1.03)	< 0.001	1.01 (1.00–1.02)	< 0.001
RV pacing burden	1.01 (1.00–1.03)	< 0.001	1.01 (1.00–1.02)	< 0.001
Paced-QRS duration	1.02 (1.01–1.04)	< 0.001		
LBBAP vs. RVSP	0.41 (0.28–0.57)	< 0.001	0.45 (0.31–0.64)	< 0.001

*HF*, heart failure; *LVEF*, left ventricular ejection fraction; *RV*, right ventricular; *LBBAP*, left bundle branch area pacing; *RVSP*, right ventricular septal pacing

Procedural and postprocedural complications are summarized in Table 3. There was no statistically significant difference in the secondary composite outcome between the two groups (3.7% vs. 5.1%, *p* = 0.65). Although one patient in the LBBAP group experienced septal perforation during the procedure, the RV pacing lead was successfully repositioned to another site. One patient in the LBBAP group and two in the RVSP group underwent lead revision during hospitalization due to lead dislodgement and threshold increases.

## Discussion

The major findings in this study were as follows: (1) LBBAP was associated with significant reduction in adverse clinical outcomes, including all-cause death, hospitalization due to HF, and upgrading to BVP, compared to accurate RVSP in patients requiring frequent RV pacing. (2) Although there were no significant differences in LVEF at baseline, the LVEF at 1 year after implantation was significantly lower in the accurate RVSP group than that in the LBBAP group.

(3) The proportion of patients with worsening TR at 1-year follow-up in the LBBAP group was significantly lower than that of the RVSP group.

(4) Even after adjusting for the RV pacing burden, baseline QRS duration, and baseline LVEF, LBBAP was associated with a significant reduction in the incidence of the primary outcome compared to accurate RVSP. (5) Although the mean procedural and fluoroscopic times were significantly longer in the LBBAP group than in the RVSP group, there was no significant difference in the incidence of procedural and postprocedural complications between the two groups.

Recently, conduction system pacing, including His bundle pacing (HBP) and LBBAP, was developed to prevent LV dysfunction resulting from pacing-induced electromechanical dyssynchrony [8–12]. Although HBP is the most physiological pacing modality, its wide adoption in clinical practice is limited by its unstable threshold, low R-wave amplitude, and atrial oversensing [13–15]. LBBAP helps to physiologically activate the ventricle by capturing the conduction system around the area of the LBB. Moreover, LBBAP has rapidly gained popularity due to its lower pacing thresholds, higher sensing amplitudes, and more stable lead positions [1–3]. LBBAP resulted in a reduction of adverse outcomes after implantation compared with conventional RV pacing. However, in these studies, RV pacing included RV septal and apical pacing. Furthermore, studies have not confirmed whether the RVSP lead was properly placed in the RV septum. Thoracic CT scans revealed that approximately 41% of cases of stylet-guided RVSP were correctly implanted in the RV septum [16–19]. Unexpected RV free wall pacing increases the incidence of cardiovascular mortality and hospitalization due to HF in patients who underwent conventional stylet-guided RVSP [6].

Recently, delivery catheters designed for pacemaker lead implantation have become widely available. A randomized controlled trial showed that delivery catheter-guided RV lead implantation could achieve more accurate RV lead placement in the RV septum and a narrower paced-QRS duration than the traditional stylet-based technique. This study is the first to use a delivery catheter in all patients to investigate the clinical impact of LBBAP compared to accurate RVSP. Although the procedural and fluoroscopic times in the LBBAP group were longer than those in the RVSP, the incidence of procedural and postprocedural complications were similar between both groups, suggesting that LBBAP is a safe and feasible pacing modality. Moreover, the LBBAP group had a shorter paced-QRS duration and paced-LVAT, which may contribute to a significant reduction in the incidence of the primary outcome compared with accurate RVSP. However, in this study, the paced-QRS duration in the RVSP group appears to be significantly longer compared with previous experience with RVSP using a similar implantation technique with a delivery catheter (130 ± 19 ms). In this study, baseline LVEF was 58.9%, which was lower than that in the previous study (64.4%). Furthermore, approximately 50% of the patients in this study had a history of HF. The baseline QRS duration was longer, and more patients had left or right bundle branch block, suggesting that the study included many patients with severe infra-Hisian block. In the case of severe infra-Hisian disease, the paced-QRS duration may not get narrowed even with septal pacing by the delivery catheter. These differences may explain the longer paced QRS duration seen in the septal pacing group of this study compared to prior studies.

The RVSP group had a 3.3% reduction in LVEF at 1-year follow-up, whereas the LBBAP group had a significantly lower reduction in LVEF compared to the RVSP group. LBBAP offers a significant advantage over accurate RVSP in a population at high risk of pacing-induced cardiomyopathy (PICM). Our results are consistent with previous studies comparing the stylet-guided RV pacing.

TR is a known complication of cardiac implantable electrical devices (CIEDs) implantation with a prevalence of up to 7–45% [20]. In a previous study, Bednarek et al. reported that 13.9% (17/122) of patients treated with LBBAP had worsening TR [21]. Moreover, Li et al. investigated the occurrence of worsening TR between the LBBAP and RVSP groups [22]. They demonstrated that the worsening TR rates at 1 and 2 years were similar between the two groups (LBBAP vs. RVSP, 1-year outcome: 15.2% vs. 17.2%; 2-year outcome: 21.6% vs. 24.6%) The rate of occurrence of worsening TR of the RVSP group in our study was similar to that in the previous studies (17.7% vs. 17.2%), while the same parameter in the LBBAP group was lower in our study (7.4% vs. 15.2%). They revealed that the distance from the electrode fixation site to the tricuspid annulus (E-T distance) of > 19 mm might be a major factor influencing LBBAP lead-related TR. Moreover, the occurrence of worsening TR in the patients with E-T distance of > 19 mm was 10 ± 4% and was similar to that of our study. In our study, the mean value of E-T distance in the LBBAP group was 25.7 ± 6.9 mm, which is well above 19 mm, and this might be the reason for the low rate of worsening TR. However, this is a relatively acute echocardiographic finding at 1-year follow-up, and chronic TR is due to decreased mobility of the tricuspid valve caused by lead adhesion, and therefore requires longer follow-up.

This study has several limitations. First, this was a single-center nonrandomized study with a relatively small sample size. Second, the decision to perform LBBAP or RVSP was left entirely to the operator’s direction. Moreover, it was not evenly distributed among operators, and certain operators who were skilled in LBBAP tended to perform LBBAP. Thus, this bias may have affected the success rate of LBBAP, procedure time, fluoroscopic time, and number of complications. Third, nine patients in whom LBBAP could not be performed had RVSP; therefore, the difference between both strategies may be underestimated.

Fourth, we did not confirm the lead position using computed tomography (CT). In this study, we performed contrast injection through delivery catheter before lead deployment and confirmed that the catheter was located at the RV septum. A previous study demonstrated that performing contrast injection through the delivery catheter either before or after lead deployment and applying the strict fluoroscopic RAO criteria could improve the success rate of accurate RVSP [23]. Although this method is promising for accurate septal pacing, it can be difficult to determine if it is a true septum based on fluoroscopic images alone in some cases, and the lead position should have been confirmed by CT after pacemaker implantation. Moreover, given that in previous study [7], 78% of leads implanted with a delivery catheter were placed in a true RV septum, it is possible that our method of using a delivery catheter may still not have led to true septal placement.

Although our results revealed that LBBAP was superior to accurate RVSP using a delivery catheter in terms of lead parameters and clinical outcomes, large prospective randomized trials with a long-term follow-up period are needed to confirm the differences in outcomes noted in this study.

## Conclusions

In patients requiring frequent RV pacing due to AVB, LBBAP was associated with reduction of adverse clinical outcomes, including all-cause death, hospitalization due to HF, and upgrading to BVP, compared with accurate RVSP using a delivery catheter.

## Supplementary Information

Below is the link to the electronic supplementary material.

Supplementary file1 (JPG 28 KB)

## Data Availability

The data that support the findings of this study are available from the corresponding author upon reasonable request.

## Abbreviations

AF: Atrial fibrillation
AVB: Atrioventricular block
BVP: Biventricular pacing
CRT: Cardiac resynchronized therapy
HF: Heart failure
ICD: Implantable cardioverter defibrillator
LAO: Left anterior oblique
LBBAP: Left bundle branch area pacing
LVAT: Left ventricular activation time
LVEF: Left ventricular ejection fraction
RVSP: Right ventricular septal pacing
TR: Tricuspid regurgitation
