# Supplementary figures and images for: Mid-term clinical outcomes of left bundle branch area pacing compared to accurate right ventricular septal pacing
Source: J Interv Card Electrophysiol. 2024 Jul 29;68(1):55–63. doi: 10.1007/s10840-024-01890-z (PMC11832786; doi:10.1007/s10840-024-01890-z)

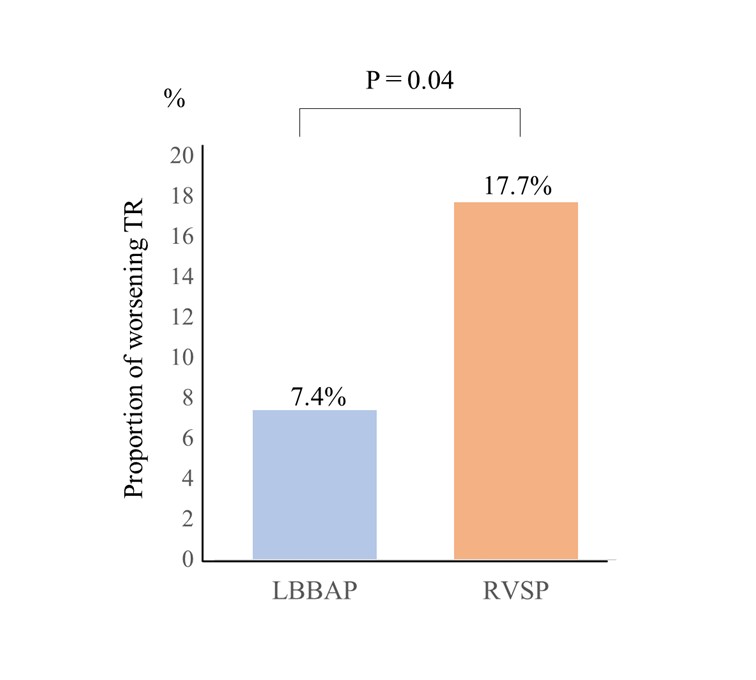

Supplement: Supplementary file 1 — Supplementary file1 (JPG 28 KB) [file 10840_2024_1890_MOESM1_ESM.jpg]
